# Psoriasis Patients are Associated with Increased Risk of New-Onset Irritable Bowel Syndrome: A Multicenter, Retrospective Cohort Study

**DOI:** 10.7150/ijms.116551

**Published:** 2025-07-24

**Authors:** Shiu-Jau Chen, Hui-Chin Chang, Shuo-Yan Gau

**Affiliations:** 1 Department of Neurosurgery, MacKay Memorial Hospital, Taipei, Taiwan.; 2 Department of Medicine, MacKay Medical College, New Taipei City, Taiwan.; 3 Evidence-based Medicine Center, Chung Shan Medical University Hospital, Taichung, Taiwan.; 4 Library, Chung Shan Medical University Hospital, Taichung, Taiwan.; 5 School of Medicine, Chung Shan Medical University, Taichung, Taiwan.; 6 Department and Graduate Institute of Business Administration, National Taiwan University, Taipei, Taiwan.; 7 Department of Pharmacology, Chung Shan Medical University, Taichung, Taiwan.; 8 Orthopedics Department, Chi-Mei Medical Center, Tainan, Taiwan.

## Abstract

**Background:** Psoriasis is a chronic systemic inflammatory disease linked to multiple comorbidities. The association between psoriasis and irritable bowel syndrome (IBS) remains insufficiently characterized.

**Methods:** We conducted a retrospective cohort study using the TriNetX US Collaborative Network. Adults with psoriasis (ICD-10-CM L40) diagnosed between 2005 and 2023 were matched 1:1 with non-psoriasis controls based on demographics, socioeconomic status, comorbidities, and healthcare utilization. IBS (ICD-10-CM K58) diagnoses within 90 days of index were excluded. Cox regression models estimated hazard ratios (HRs) with 95% confidence intervals (CIs). Sensitivity analyses varied wash-out periods, follow-up durations, and exposure definitions.

**Results:** After matching (n=256,550 per group), people with psoriasis were associated with a higher risk of IBS (HR 1.244, 95% CI 1.168-1.325) in the 15-year follow-up model while comparing with non-psoriasis controls. Subgroup analyses validated elevated risks among both age groups (e.g., age ≥65 years: HR 1.325, 95% CI 1.167-1.505) and sexes (female: HR 1.291, 95% CI 1.197-1.393). All sensitivity models yielded consistent results.

**Conclusion:** Psoriasis is independently associated with an increased risk of subsequent IBS. Routine gastrointestinal symptom screening in psoriasis patients may improve comprehensive care.

## Introduction

Psoriasis is a chronic immune-mediated dermatologic condition affecting approximately 2-3% of the global population 1. Beyond cutaneous involvement, psoriasis is associated with systemic comorbidities, including increased risks of inflammatory, psychiatric, and gastrointestinal diseases 2-5. In particular, psoriasis has been significantly linked to inflammatory bowel diseases (IBD), with meta-analyses indicating a 1.7-fold increased odds of ulcerative colitis and a 2.5-fold increased odds of Crohn's disease, supporting the concept of a gut-skin axis driven by chronic systemic inflammation 6.

Irritable bowel syndrome (IBS), a prevalent disorder of gut-brain interaction affecting approximately 11% of the global population, is characterized by recurrent abdominal pain and altered bowel habits 7. IBS frequently coexists with psychological distress, with about one-third of patients experiencing anxiety or depression 8. This association highlights the role of stress and systemic factors in the manifestation of the disorder. Although IBS patients typically do not exhibit the overt intestinal inflammation seen in IBD, the condition has been linked to subtle immune dysregulation, alterations in the gut microbiota and increased intestinal permeability 9, 10.

The co-occurrence of psoriasis with gastrointestinal diseases other than IBD has received comparatively less attention. It is biologically plausible that psoriasis and IBS could be linked via shared immunological pathways or risk factors. Both conditions have been associated with alterations in the gut microbiome and immune profiles. A small case-control study suggested a high prevalence of IBS in psoriasis patients 11; however, large scale studies in real-world setting are lacking.

Given the systemic pro-inflammatory milieu of psoriasis and its overlap with pathways implicated in functional bowel disorders, we hypothesized that psoriasis is associated with an increased risk of developing IBS. We therefore conducted a large multicenter retrospective cohort study using a federated electronic health record network to investigate the risk of IBS among patients with psoriasis compared to matched controls. This study aimed to elucidate the association between psoriasis and IBS, explore subgroup differences, and advance understanding of the gut-skin axis in immune-mediated disease.

## Methods

### Study design and data source

We conducted a retrospective cohort study using the TriNetX US Collaborative Network, a federated database of de-identified electronic health records from 69 healthcare organizations across the United States. The research network has been widely applied in the field of health economics and outcomes research 12, 13. The study followed STROBE guidelines, and the Institutional Review Board of Chung Shan Medical University Hospital waived the need of informed consent of this study (CS1-25002).

### Population and outcome definition

**Figure 1:**
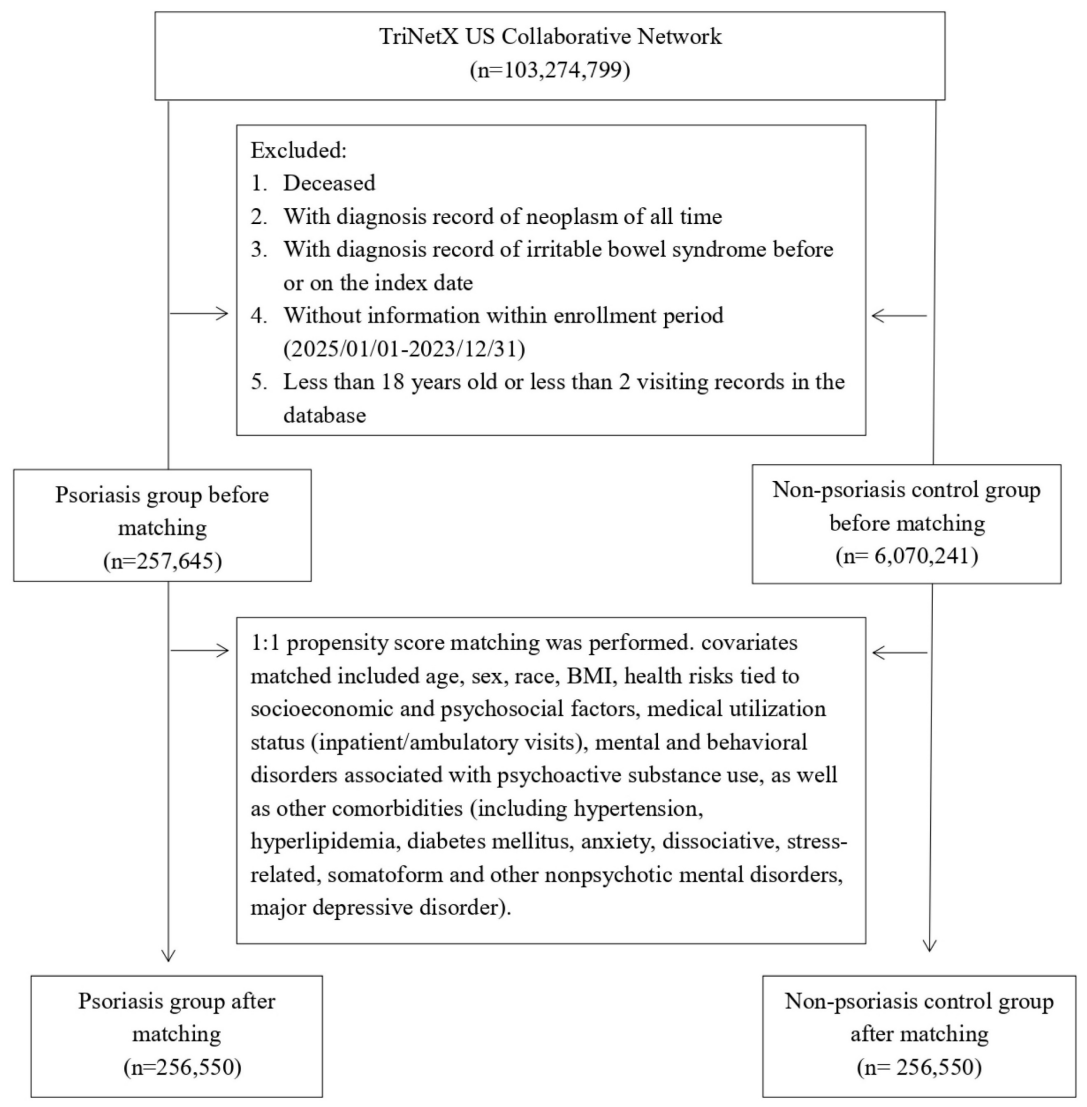
Patient selection flowchart.

The psoriasis cohort included adult patients (≥ 18 years) with at least one recorded diagnosis of psoriasis (ICD-10-CM code L40) between January 1, 2005, and December 31, 2023. The index date was defined as the first psoriasis diagnosis. The primary outcome was incident IBS occurring at least 90 days after the index date to reduce reverse causation. We excluded individuals with a prior diagnosis of IBS (ICD-10-CM code K58), any malignant neoplasm, or those who died on or before the index date. The control cohort was composed of patients without psoriasis, selected from the same network (**Figure 1**). Controls were required to have a general medical examination (ICD-10-CM code Z00) to establish an index date and were matched 1:1 to psoriasis patients based on similar inclusion criteria. In this study, a diagnosis of IBS (ICD-10-CM code K58) was used to define the outcome of interest. Follow-up for each patient ended at the time of this diagnosis, which served as the censoring point. Detailed information of used ICD-10-CM codes were presented in **Table S1**.

### Matching and covariates

Propensity score matching was performed to control for baseline confounders. Covariates included age, sex, race, BMI (≥ 25 vs < 25 kg/m²), socioeconomic and psychosocial risk factors (ICD-10 Z55-Z65), healthcare utilization, substance use disorders (ICD-10 F10-F19), and comorbidities such as hypertension, hyperlipidemia, diabetes, depression, and anxiety. The variables used for matching were selected because previous research indicates they may be linked to IBS, making them likely confounding factors that could influence the study's results. The 1:1 nearest-neighbor matching used a caliper of 0.1 standard deviations. Balance was assessed using standardized mean differences (SMD, with the value of < 0.1 indicated adequate matching).

### Statistical analysis

The Analytic tool of TriNetX Research Network, Python (version 3.12.7) and Microsoft Excel 2019 was used for main analysis and figure generation. Descriptive statistics compared baseline characteristics pre- and post-matching. Hazard ratios (HRs) with 95% confidence intervals (CIs) were calculated in each analysis. Cox proportional hazards regression was performed using the TriNetX built-in analytics system, which computes HRs and CIs based on time-to-event data aggregated from electronic health records. A cumulative incidence plot was presented, with the x-axis representing time since the index date (in months), and the y-axis showing the cumulative incidence of IBS. Differences between curves were tested using the log-rank test, with statistical significance defined as p < 0.05. Subgroup analyses stratified by age (< 65 vs ≥ 65 years) and sex, and multiple sensitivity analyses were performed to test robustness across washout periods, follow-up lengths, matching strategies, and stricter psoriasis definitions (Details reported in **Table S2**).

## Results

### Baseline characteristics

Before matching, 257,645 psoriasis patients and 6,070,241 non-psoriasis controls met inclusion criteria. After 1:1 propensity score matching, 256,550 patients remained in each group (**Table 1**). Baseline characteristics were notably imbalanced pre-matching, with psoriasis patients showing higher age, greater proportions of White individuals, and a higher burden of cardiometabolic and psychiatric comorbidities. After matching, covariate balance was achieved (all standardized mean differences < 0.1).

### Risk of IBS and sensitivity analysis

**Figure 2:**
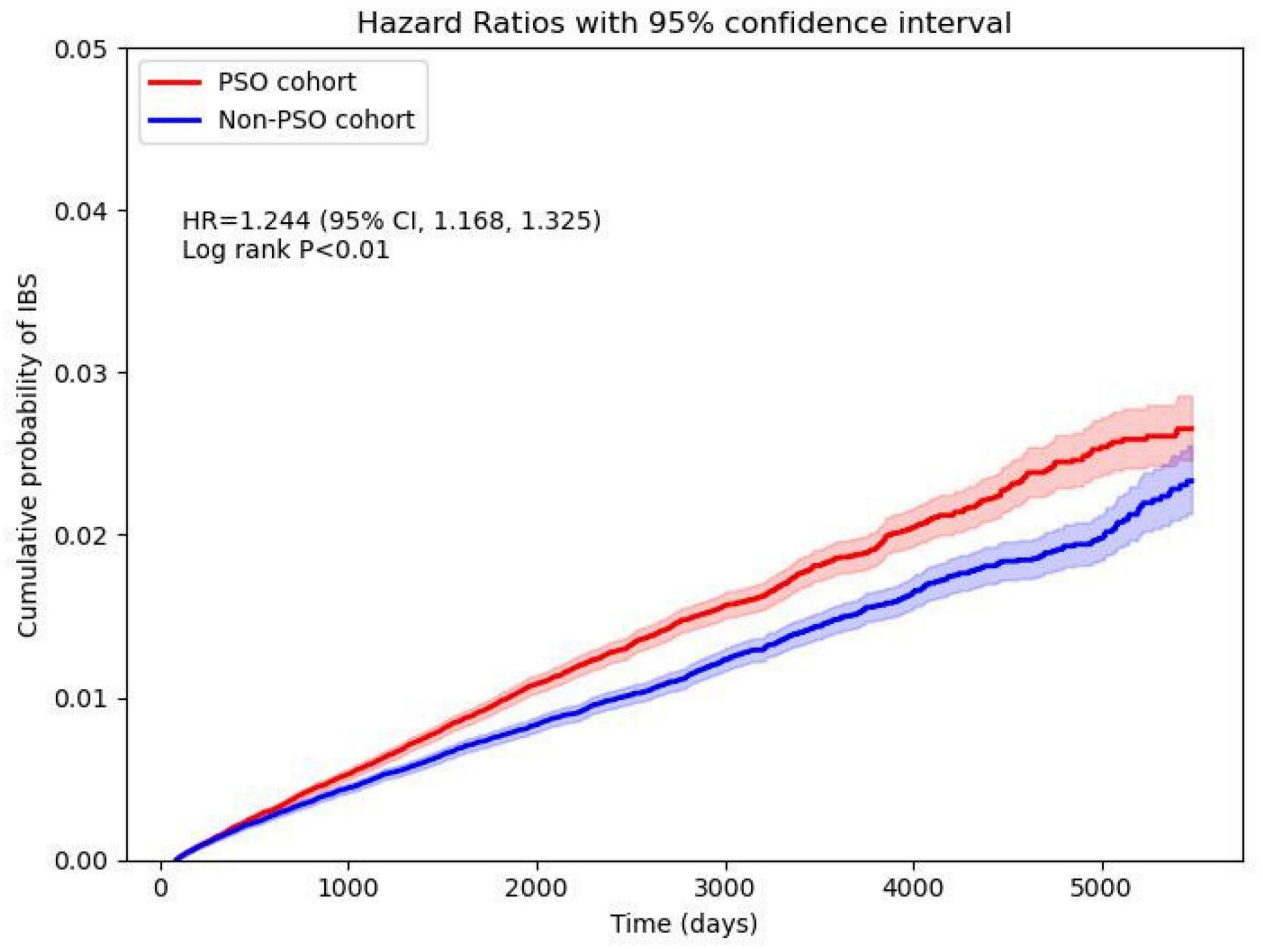
Cumulative probability curve of irritable bowel syndrome risk in psoriasis and non-psoriasis cohorts.

**Figure 3:**
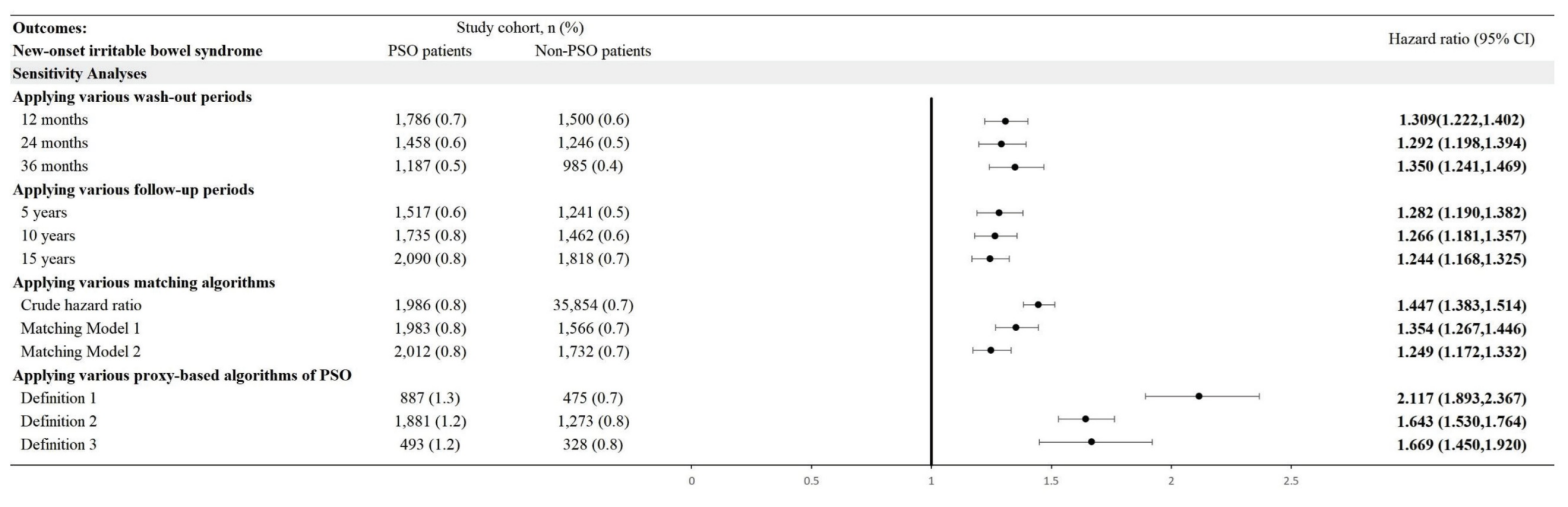
Risk of irritable bowel syndrome in various sensitivity mode.

In the 15-year follow-up, the risk for psoriasis patients developing new onset IBS, while comparing with non-psoriasis controls, was 1.244-fold (95% CI, 1.168-1.325) (**Figure 2**). Robustness of the primary finding was confirmed in multiple sensitivity analyses (**Figure 3**). We varied the IBS wash-out period to 12, 24, and 36 months to address potential misclassification of pre-existing IBS. HRs remained statistically significant and consistent across all time frames (e.g., HR at 12 months = 1.309, 95% CI 1.222-1.402; at 36 months = 1.350, 95% CI 1.241-1.469), suggesting that reverse causation or latent cases did not explain the observed association. When altering the maximum follow-up duration yielded consistent results. With follow-up truncated at 5 years, the HR for IBS in the psoriasis group was 1.282 (95% CI, 1.190-1.382); with 10 years, HR was 1.266 (95% CI, 1.181-1.357)—supporting the presence of short- and intermediate-term risk elevations. Alternative analytic approaches of matching covariates were also tested. In the unmatched crude cohort, the HR for IBS was 1.447 (95% CI, 1.383-1.514). Additionally, we applied strict definitions of psoriasis to address potential misclassification bias. When psoriasis exposure was defined as ≥ 2 visiting records plus an inpatient encounter (Algorithm 1), the HR for IBS was 2.117 (95% CI, 1.893-2.367). Among those receiving systemic corticosteroids (Algorithm 2), the HR was 1.643 (95% CI, 1.530-1.764); for those treated with biologics (Algorithm 3), the HR was 1.669 (95% CI, 1.450-1.920). Since IBD and IBS exhibit overlapping symptoms, and IBD has a well-established link to psoriasis, there's a risk that the observed relationship between psoriasis and IBS could be influenced by the presence of IBD. After exclusion of IBD patients, the association between psoriasis and IBS remained statistically significant, with a hazard ratio of 1.312 (95% CI, 1.242-1.386).

### Stratified analyses

Subgroup analyses confirmed that the association was consistent across age and sex strata (**Table 2**). Among individuals under 65 years, the adjusted HR for IBS was 1.254 (95% CI, 1.165-1.351), while in those aged ≥ 65, the HR was 1.325 (95% CI, 1.167-1.505). The HR among female patients was 1.291 (95% CI 1.197-1.393), and 1.155 (95% CI 1.012-1.318) among males.

## Discussion

In this large, multicenter retrospective cohort study using an electronic health record derived real-world database, we found that patients with psoriasis had a significantly increased risk of developing IBS compared to matched controls, with a hazard ratio of 1.244 (95% CI, 1.168-1.325) in 15-year follow-up period. To our knowledge, this is the first large-scale study to quantify this association longitudinally, using a rigorously matched population and real-world data from a diverse US patient cohort.

Our findings align with prior smaller-scale studies, including the case-control study by Unal et al., which reported a substantially higher prevalence of IBS in psoriasis patients (36.9%) compared to controls (12.6%) 11. Although their effect size was larger, differences in study design likely explain the disparity: their study used direct symptom screening (Rome III criteria), whereas ours relied on diagnostic coding from clinical encounters. Our design likely captured moderate-to-severe IBS that came to clinical attention, possibly underestimating subclinical or mild cases. Nevertheless, both studies support the concept of a psoriasis-IBS link. Importantly, our study demonstrated temporal sequencing, with psoriasis preceding IBS onset in all cases. The association persisted after rigorous propensity score matching and covariate adjustment, including control for psychiatric comorbidities such as depression and anxiety, which are independently associated with IBS. This suggests that shared risk factors alone are insufficient to account for the observed relationship, pointing toward a potential pathophysiological connection between the two conditions.

The concept of a gut-skin axis has gained considerable traction, particularly in the context of immune-mediated diseases. Psoriasis shares immunological pathways with IBD, including Crohn's disease and ulcerative colitis, notably through dysregulation of the IL-23/Th17 axis 14. While IBS lacks the overt mucosal inflammation of IBD, evidence suggests that low-grade immune activation may underlie a subset of IBS cases 10, 15. Systemic inflammation is a hallmark of psoriasis, driven by pro-inflammatory cytokines such as TNF-α, IL-17, and IL-23 16, which have known effects on gut permeability and neuroenteric signaling 17, 18. These cytokines may “prime” the gut environment for IBS-like symptoms by disrupting mucosal homeostasis or altering the enteric nervous system. Our findings extend the gut-skin axis model by implicating not only classical inflammatory bowel diseases but also functional disorders like IBS in the comorbidity spectrum of psoriasis.

Another potential mechanistic link between psoriasis and IBS involves the gut microbiome. Psoriasis has been associated with alterations in gut microbiota composition, particularly a reduction in anti-inflammatory bacterial genera 19. For instance, a recent case-control study noted significantly elevated gut interleukin-1α levels and altered gut microbial composition in psoriasis patients compared to controls​ 20. Such dysbiosis may lead to changes in microbial metabolites—such as imbalanced short-chain fatty acids—which can affect gut motility and visceral sensitivity 21. Similarly, patients with IBS often exhibit microbial imbalance, and in some cases, fungal overgrowth 10. The "leaky gut" phenomenon—characterized by increased intestinal permeability noted in IBS—may permit the translocation of microbial products into systemic circulation, fueling chronic inflammation and potentially exacerbating psoriasis, forming a self-perpetuating inflammatory loop 22. An altered microbiome in individuals with psoriasis may predispose them to IBS-like symptoms by promoting gas production, disrupting bile acid metabolism, or activating toll-like receptors, thereby contributing to visceral hypersensitivity 23, 24.

Moreover, the brain-gut-skin axis provides an integrative framework, wherein psychological stress can concurrently trigger neuroimmune responses in both the gastrointestinal tract and the skin 25, 26. Stress-induced activation of the hypothalamic-pituitary-adrenal (HPA) axis, sympathetic nervous system, and subsequent cortisol release can impair skin barrier function, alter gut motility, and provoke IBS flare-ups and psoriasis exacerbations, linking both conditions through a neuro-immuno-endocrine pathway 27, 28.

Several limitations warrant consideration. First, although the longitudinal design supports temporal inference, this remains an observational study and cannot establish causality. Despite comprehensive matching and adjustment, residual confounding by unmeasured variables (e.g., stress levels, dietary habits, physical activity) may exist 29. Second, our reliance on ICD-10 codes for psoriasis and IBS introduces the potential for misclassification. Some patients may have been miscoded, and we lacked data on standardized IBS diagnostic criteria (e.g., Rome IV). However, such misclassification would likely be non-differential between groups, biasing estimates toward the null. Third, the cohort was drawn from healthcare-engaged individuals within the US, and findings may not generalize to those with limited healthcare access or in other countries. The sample was predominantly White, which mirrors psoriasis epidemiology in the US, but limits extrapolation to other racial/ethnic groups. Fourth, psoriasis severity could not be precisely assessed. Although we used proxy markers such as inpatient encounters and systemic therapy, we lacked standardized measures like the Psoriasis Area and Severity Index (PASI). It remains possible that IBS risk is higher in more severe cases, and future studies should explore this gradient.

## Conclusion

From a clinical standpoint, our findings underscore the importance of recognizing IBS as a potential comorbidity in patients with psoriasis. Clinicians should maintain a high index of suspicion for IBS in psoriasis patients who report chronic abdominal pain, bloating, or altered bowel habits—particularly when IBD has been ruled out. Dermatologists and gastroenterologists should consider interdisciplinary collaboration in such cases to optimize patient care. Early diagnosis and management of IBS may significantly improve quality of life in this population.

## Supplementary Material

Supplementary figures and tables.

## Figures and Tables

**Table 1:** Baseline characteristics

	Before matching			After matching^a^		
	PSO cohort (n=257,645)	Non-PSO control cohort (n=6,070,241)	SMD	PSO cohort (n=256,550)	Non-PSO control cohort (n=256,550)	SMD
**Age at index**						
Mean±SD	46.3 ± 17.9	38.2 ± 20.2	0.43	46.3 ± 17.9	46.3 ± 19.0	0.00
**Sex**						
Male	112007 (43.7)	2565805 (42.5)	0.02	112007 (43.7)	109968 (42.9)	0.02
Female	131997 (51.5)	3125056 (51.7)	0.01	131997 (51.5)	131897 (51.4)	0.00
Unknown	12546 (4.9)	352472 (5.8)	0.04	12546 (4.9)	14685 (5.7)	0.04
**Race, n (%)**						
White	177766 (69.3)	3480142 (57.6)	0.24	177766 (69.3)	177724 (69.3)	0.00
Black or African American	16471 (6.4)	892867 (14.8)	0.27	16471 (6.4)	16604 (6.5)	0.00
Asian	10383 (4.0)	295999 (4.9)	0.04	10383 (4.0)	11121 (4.3)	0.01
Native Hawaiian or Other Pacific Islander	1924 (0.8)	38613 (0.6)	0.01	1924 (0.8)	1475 (0.6)	0.02
American Indian or Alaska Native	865 (0.3)	19756 (0.3)	0.00	865 (0.3)	731 (0.3)	0.01
Unknown Race	36880 (14.4)	962041 (15.9)	0.04	36880 (14.4)	36185 (14.1)	0.01
Other Race	12261 (4.8)	353915 (5.9)	0.05	12261 (4.8)	12710 (5.0)	0.01
**BMI, n (%)**						
≥ 25 (kg/m^2^)	73013 (28.5)	1498134 (24.8)	0.08	73013 (28.5)	73301 (28.6)	0.00
**Medical Utilization Status, n (%)**						
Visit: Ambulatory	160824 (62.7)	3555817 (58.8)	0.08	160824 (62.7)	161066 (62.8)	0.00
Visit: Inpatient Encounter	37478 (14.6)	739908 (12.2)	0.07	37478 (14.6)	37298 (14.5)	0.00
**Socioeconomic status, n (%)**						
Persons with potential health hazards related to socioeconomic and psychosocial circumstances	3088 (1.2)	87661 (1.5)	0.02	3088 (1.2)	2818 (1.1)	0.01
**Lifestyle, n (%)**						
Mental and behavioral disorders due to psychoactive substance use	22171 (8.6)	356705 (5.9)	0.11	22171 (8.6)	22058 (8.6)	0.00
**Comorbidities, n (%)**						
Major depressive disorder	5123 (2.0)	91264 (1.5)	0.04	5123 (2.0)	4796 (1.9)	0.01
Anxiety, dissociative, stress-related, somatoform and other nonpsychotic mental disorders	29268 (11.4)	536049 (8.9)	0.08	29268 (11.4)	28911 (11.3)	0.00
Hyperlipidemia	26737 (10.4)	476675 (7.9)	0.09	26737 (10.4)	26472 (10.3)	0.00
Essential hypertension	43810 (17.1)	803107 (13.3)	0.11	43810 (17.1)	43607 (17.0)	0.00
Diabetes mellitus	19670 (7.7)	330822 (5.5)	0.09	19670 (7.7)	19345 (7.5)	0.00

PSO, psoriasis; SMD, standardized mean difference; SD, standardized difference ^a^ The covariates matched included age, sex, race, BMI, health risks tied to socioeconomic and psychosocial factors, medical utilization status (inpatient/ambulatory visits), mental and behavioral disorders associated with psychoactive substance use, as well as other comorbidities (including hypertension, hyperlipidemia, diabetes mellitus, anxiety, dissociative, stress-related, somatoform and other nonpsychotic mental disorders, major depressive disorder).

**Table 2:** Stratification analysis of IBS risk in PSO patients in 15-year follow-up

	Cases occurring new-onset IBS		
Subgroups	PSO cohort No. of outcome events (%)	Control cohort No. of outcome events (%)	HR (95% CI)^a^
**Age at index date**			
18-64 years old	1,527 (0.9)	1,295 (0.7)	**1.254 (1.165,1.351)**
≥ 65 years old	521 (0.7)	438 (0.6)	**1.325 (1.167,1.505)**
**Sex**			
Male	463 (0.4)	418 (0.4)	**1.155 (1.012,1.318)**
Female	1,443 (1.1)	1,244 (1.0)	**1.291 (1.197,1.393)**

^a^ The covariates matched included age, sex, race, BMI, health risks tied to socioeconomic and psychosocial factors, medical utilization status (inpatient/ambulatory visits), mental and behavioral disorders associated with psychoactive substance use, as well as other comorbidities (including hypertension, hyperlipidemia, diabetes mellitus, anxiety, dissociative, stress-related, somatoform and other nonpsychotic mental disorders, major depressive disorder).
