# Supplementary material for: Psoriasis Patients are Associated with Increased Risk of New-Onset Irritable Bowel Syndrome: A Multicenter, Retrospective Cohort Study
Source: Int J Med Sci. 2025 Jul 24;22(13):3422–8. doi: 10.7150/ijms.116551 (PMC12320781; doi:10.7150/ijms.116551)
Supplement: Supplementary file 1 — Supplementary figures and tables. [file ijmsv22p3422s1.pdf]

Supplementary Files

Table S1. Utilized administrative codes [a]

**Description**	**ICD-10-CM codes**
Study population	
Psoriasis	ICD-10-CM: L40
Outcome event	
Irritable bowel syndrome	ICD-10-CM: K58
Covariates and other definitions	
Essential hypertension	ICD-10-CM: I10
Hyperlipidemia	ICD-10-CM: E78.5
Diabetes mellitus	ICD-10-CM: E08-E13
Major depressive disorder	ICD-10-CM: F33
Anxiety, dissociative, stress-related, somatoform and other nonpsychotic mental disorders	ICD-10-CM: F40-F48
Persons with potential health hazards related to socioeconomic and psychosocial circumstances	ICD-10-CM: Z55-Z65
Mental and behavioral disorders due to psychoactive substance use	ICD-10-CM: F10-F19
Encounter for general examination	ICD-10-CM: Z00

[a]ICD-10-CM: International Classification of Diseases, Tenth Revision, Clinical Modification

Table S2. Description of all applied sensitivity analysis models in Figure 3

Models	Description
**Applying different wash-out and follow-up period after index date**	
Wash-out periods: 12 months/24 months/36 months after index date	Outcome event occurs within the set wash-out period will be excluded from further analysis. In all sub-analyses of this model, the follow-up period was set as 15 years.
Follow-up periods: 5 years /10 years /15 years after index date	Only the outcome event occurs within the set follow-up time will be counted. In all sub-analyses of this model, the wash-out period was set as 3 months.
**Applying different matching covariates**	
Crude model	Hazard ratio was calculated based on the population without propensity score matching
Matching model 1	Matching covariates include age at index and sex
Matching model 2	Matching covariates include age at index, sex, race, socioeconomic status, medical utilization status
**Applying various proxy-based psoriasis (PSO) definition**	
Algorithm 1	Only patients diagnosed PSO with more than 2 visit records and was limited to have at least one impatient visit were included as PSO group in this model.
Algorithm 2	Only patients diagnosed PSO with more than 2 visit records and with the record of systemic corticosteroid (ATC code: H02) were included as PSO group in this model.
Algorithm 3	Only patients diagnosed PSO with more than 2 visit records and with the record of biologics for PSO treatment, including etanercept (RxNorm: 214555), infliximab (RxNorm: 191831), adalimumab (RxNorm: 327361), certolizumab pegol (RxNorm: 709271), ustekinumab (RxNorm: 847083), secukinumab (RxNorm: 1599788), ixekizumab (RxNorm: 1745099),

	brodalumab (RxNorm: 1872251), guselkumab (RxNorm: 1928588), tildrakizumab (RxNorm: 2053436), were included as PSO group in this model.
